# Adverse reactions to food: the female dominance – A secondary publication and update

**DOI:** 10.1186/s40413-017-0174-z

**Published:** 2017-12-27

**Authors:** Sheriene Moussa Afify, Isabella Pali-Schöll

**Affiliations:** 1 Comparative Medicine, The Interuniversity Messerli Research Institute of the University of Veterinary Medicine Vienna, Medical University Vienna and University Vienna, Vienna, Austria; 2 Laboratory Medicine and Immunology Department, Faculty of Medicine, Menoufia University, Menoufia, Egypt; 3 Institute of Pathophysiology and Allergy Research; Center of Physiology, Pathophysiology and Immunology, Medical University Vienna, Vienna, Austria

**Keywords:** Female, Food allergy, Food intolerance, Fructose, Gender, Histamine, Lactose, Women

## Abstract

Gender-specific differences are evident in food intolerance and allergy. In this review, we will highlight and summarize the dissimilarities in prevalence of adverse food reactions, focusing on IgE-mediated food allergies and intolerances regarding frequency of symptoms and predisposing factors. After puberty, females suffer more frequently from food-related symptoms than males. Several factors may be responsible for this observation, for example hormonal effects, gender-specific behavior, perception of risk, or intake of medications. In this context, concrete studies related to adverse food reactions are still lacking.

## Background

Adverse food reactions affect men as well as women. However, for most of the associated diseases (allergies, intolerances), an imbalance mainly in the direction of a dominance of female patients is observed. In this article we discuss the prevalence of food intolerance conditions and IgE-mediated food allergies, but refrain from including non-IgE mediated allergies or sensitization (i.e., simple presence of specific IgE without correlated clinical symptoms evaluated or reported). We used the search terms “food allergy” AND gender, “food allergy” AND female, “food intolerance” AND gender, as well as “food intolerance” AND female in PubMed. Foremost, the possible factors related to the female dominance in such diseases have been extracted.

## Prevalence of adverse food reactions

### Allergies

Interestingly, it was noted that in childhood and adolescence (before puberty, till 15 years) boys are more affected often by general atopic conditions (skin reactions against one or more allergens [1]) as well as asthma and food allergies [2] than girls. Later in life (young adult group; 13–21 years), females are significantly more affected by food-induced complaints (24% vs. 14% in males) [3], as assessed by a questionnaire-based survey. Other studies have confirmed this observation (Table 1), and show that the ratio in prevalence of food intolerance between females and males after puberty is 60:40 [4, 5]. For instance, in a more recent report, 20,686 cases were included in the time period between 2007 and 2010, from which the prevalence of self-observed food intolerance in females was 11.1% compared to 8.21% in males with an overall prevalence of 8.96% in the studied population [6], and among Mexican people, basically 37.8% of female participants vs. 25.2% of male participants have reported to suffer from an adverse food reaction [7]. In this more antigen-focused questionnaire-based survey with 1238 adult participants from Mexico, an overall prevalence of self-reported wheat or gluten intolerance of about 11.9%, with a significant female predominance (14.6%) compared to male participants (8.0%) was revealed [7]. Interestingly, females suffered equally often from hay fever as males (about 63% of survey participants), which reflects a female predominance especially in food allergies [8].

**Table 1:** Prevalence of food adverse reactions in women and men found in different studies

Criteria	Female Male	Number of surveyed subjects	Study population^a^	Age (years)	Reference
Food allergic patients	65.18^b^ 34.82	17,528	Systematic search of PubMed literature	Adults (> 18 years)	[64]
Self-reported food adverse reactions	24^c^ 14	1488	1	13–21	[3]
Self-reported food adverse reactions (Interview)	20.1^b^ 13.4	1.943	1	From 14 years	[65]
Norwegian National Reporting System and Register	60^d^ 40	Ca. 6500 health care professionals/year report cases	2	Adolescents	[4]
Food-dependent exercise-induced anaphylaxis	2^e^ 1	199	1	15–35	[30]
Self-observed food adverse reactions	11.1^c^ 8.21	20,686	1	Adults	[6]
Self-reported food adverse reactions	37.8^c^ 25.2	1238	1	Adults	[7]
Self-reported food adverse reactions; Positive skin-test with at least 1 allergen	27.5^c^ 14; 27.5^c^ 22.7	1537	1	Adults	[5]
Self-reported food adverse reactions	2^e^ 1	1253	1	18–25	[66]
Physician-diagnosed food allergy in parents of food allergic children	13.3^c^ 8.9	1.252 mothers, 1.225 fathers	1 and 2	30–39	[67]
Electronic health record data for food allergy and intolerances, validated for peanut allergy by RAST and ImmunoCAP	4.2^c^ 2.9	2,714,851	2	No limitation	[12]

^a^Study population: 1 = population based/community setting, 2 = hospital based

^b^Percentage among allergic patients

^c^Percentage among study population

^d^Percent

^e^Ratio

A study from Kyoto compared only females at different ages regarding the prevalence of self-reported food intolerance [9]. This study has shown nearly the same results for adolescent (18–24 years) and elder women (< 50 years), with a prevalence of 8.2% and 8.9%, respectively. This was confirmed by other data about occurrence of food allergy in an adult population, which showed that both, adolescent (18–29 years; 28.4%) and elderly people (70–79 years; 21.1%) were sensitized against food allergens ([10], review [11]).

In a recent paper, using data from an electronic health records (EHR) allergy module from the Greater Boston area, which is composed of multiple community and specialty hospitals, food allergy and intolerance data were analyzed among 2,714,851 patients [12]. Also here, among the overall 3.6% patients affected by adverse food reactions, female sex dominated significantly with 4.2% over male with 2.9%.

This female dominance might have more far-reaching implications for further generations, because Arshad et al., revealed in their paper that in the Isle of Wight Birth Cohort, maternal allergy increased the risk for asthma, eczema, atopy and total IgE in girls but not in boys, whereas paternal allergy increased the risk in boys [8]. This has implications for childhood allergy prediction and prevention, because if there is a greater prevalence of female allergy with higher impact on girls, the effect might be multiplied. Ideally, this observation may facilitate some pattern of preventing allergy. However, more multigenerational studies are needed to assess this effect and whether there are epigenetic effects regarding the risk of allergy and asthma in subsequent generations, which may also depend on the sex of the child [13].

### Intolerances

Intolerances also clearly showed gender-specific variations with females more affected than males. The most important examples are histamine intolerance (about 1% of population, from which 80% were females [14]) and fructose intolerance [15]. Yet, in lactose intolerance, the available data are not conclusive about whether females are more affected than males [16], or whether they are equally affected [17].

## Pathophysiology

Adverse food reactions are divided according to their pathophysiology into immune-mediated conditions (for example, IgE-mediated allergy or coeliac disease) and non-immune-mediated conditions (mainly intolerances due to enzyme- or transporter deficiency) [18].

### Allergies

Food allergy — genetically predetermined, but not directly inherited — is best described as an immediate IgE-mediated reaction [19, 20], dominated by a Th2-milieu. These cells are characterized by the liberation of cytokines IL-4, IL-13 and IL-5, which consequently stimulate class switching in B-cells in the direction of IgE-antibodies production [21]. IgE-antibodies bind through high-affinity receptors to effector cells (i.e. mast cells in the tissue and basophilic leukocytes in blood). By subsequent ingestion of the specific food antigen, cross-linking of several IgE-antibodies at effector cells leads to degranulation of these cells and liberation of several mediators e.g. histamine, heparin and leukotriene, which induce allergy symptoms. The interactions of IgE with its receptor, as well as of the antigen with its IgE-antibodies were recognized as high-affinity bindings [22]. Therefore, specific IgE-antibodies are regarded as useful parameters in diagnosis of type-I allergies.

To date, two different routes for initiation of IgE-mediated true food allergies have been described: 1) primary oral sensitization through food intake, or 2) secondary cross-reaction, in which the patient is sensitized against inhalative allergens (e.g., pollens) and produces IgE-antibodies that react to homologous proteins in food (like nuts, apples, and carrots).

### Intolerances

Enzyme- or transporter defects are the most common etiologies for food intolerances [23]. Histamine intolerance for example is characterized by inadequate histamine degradation through the enzyme diaminoxidase (DAO) in the small intestine. This results in excess amounts of histamine (endogenous as well as exogenous from food), which leads to development of symptoms like migraine, flush, erythema, itching, rhinorrhea and/or gastrointestinal upset.

Another example is lactose intolerance due to lactase enzyme deficiency in small intestinal epithelium. This results in deficient digestion of milk sugar in the small intestine. Excess amount of lactose reaches the large intestine, where it gets fermented by the intestinal bacteria with production of gases e.g. methane and hydrogen (H_2_), causing flatulence and abdominal pain. In addition, short chain fatty acids and osmotically active substances are produced in high amounts, leading to diarrhea. A small study described an association between lactose intolerance and premenstrual tension syndrome, as well as mental depression especially in females. A possible theory is that high concentrations of lactose interfere with tryptophan and serotonin metabolism, which are crucial for mood control [24].

Similar symptoms could occur in fructose intolerance, in which fructose transporter (Glucose-Transporter-5, GLUT-5) is absent or deficient. This leads to inability of the small intestine to take up fructose, as a result fructose accumulates in the large intestinal lumen. Patients in such condition suffer from similar symptoms like in lactose intolerance, however, depression is more likely to occur in association with fructose intolerance because of tryptophan deficiency [25].

## Symptoms

### Allergies

Symptom elicitation is not dose-dependent in true allergy; this means that even upon intake of small amounts of allergen, symptoms of varying intensity up to life-threatening reactions can arise, also within the same patient at different time points of allergen encounter. Symptoms of IgE-mediated food allergy can occur anywhere along the digestive tract, or manifest systemically.

Marklund and colleagues have shown that in the adult population (13–21 years) about 52% of both male and female with known food-associated reactions mainly suffer from OAS. In contrast, more women than men are affected by gastrointestinal and skin reactions, especially urticaria (5.2% vs. 1.1%) and in addition by migraine (16.9% vs. 3.4%) [3]. In general, a significant positive association was proven for food-associated reactions and physicians-diagnosed hay fever, asthma, atopic dermatitis and self-reported urticaria.

In the previously mentioned study from Japan comparing food-allergic females at different ages, no age-dependent differences in the affected organs have been found: in order of frequency these were skin, mouth cavity, digestive tract, respiratory tract and anaphylactic shock [9]. The latter had happened in about 5% of all food-allergic patients. These severe systemic anaphylactic reactions can present as life-threatening symptoms like severe hypotension, tachycardia, and generalized urticaria up to circulatory shock. Such severe forms of food allergy were rare 35 years ago, however, in the meantime they represent the most common cause of anaphylaxis in emergency departments in the USA [26–28]. In a study with patients between 1 and 79 years (mean age = 37 years), a female predominance also of severe allergic reactions was obvious (62% of cases). For the identified offending substances in anaphylaxis, food remains at the top of the list (22% of cases), followed by medications (11%) and exercise (5%) [29].

In a special combined form, food-dependent exercise-induced anaphylaxis can occur. This condition is most likely to occur in females between 15 and 35 years (ratio female: male = 2:1) [30]. In such conditions, reactions happen when the patient is exposed to a strenuous situation such as physical training within 2–4 h after food intake (e.g. shrimps, wheat) (review [31]).

In general, females suffering from allergy-associated symptoms describe significantly more deterioration of health-related quality of life than males [3].

### Intolerances

In contrast to allergies, severe generalized symptoms are rare to happen in intolerances. Typical symptoms are distension, abdominal cramps and pain because of gas production, as well as diarrhea because of laxative effects of short-chain fatty acids and other osmotically active substances. Migraine can additionally occur as a symptom in case of histamine intolerance. In fructose intolerance, depression often is observed as associated symptom [5].

In case of food intolerances, symptoms usually are dependent on the amount of the offending food. This means that small quantities are still tolerated by patients; however, the exact dosage differs from patient to patient and has to be determined individually.

## Triggering food

### Allergies

The most common allergy-triggering foods in children are milk, egg, peanuts, walnuts, fish and shellfish, and in adults shellfish, peanuts, walnuts, fish, milk and egg are important [26]. If pollen-associated as well as intolerance-inducing food is included, the most frequent elicitors were nuts (39%), fruits and berries (35%), peanut (32%), almond (22%), tomato (19%) carrot (16%), lactose (12%), vegetables (10%), crustacean (9%), soy (7%), milk (7%), fish (5%) and egg (5%) in the above-mentioned adolescent patient group [3]. Gender variations in offending food are specifically observed with fruits and berries, which are more commonly reported among females (44%) compared to males (24%) as triggers of adverse food reactions, whereas males reported peanuts as a trigger of allergic reactions more often than females (43% vs. 27%).

Schäfer and colleagues correspondingly described general female predominance (27.5%) in positive skin tests for food allergens compared to males (22.7%), especially evident for peanuts (20.4% vs. 15.2%) and pollen-associated food like celery (17.2% vs. 12.1%) [32]. Out of 18 recorded possible allergens, 11 were reported more often by females to cause reactions, with statistically significant difference in case of citrus-fruits. More research is required to clarify this phenomenon.

According to a study from Japan, comparison of younger and older females with known food allergy came out with no age-related difference concerning ranking of allergy-triggering food; fruits, shellfish, fish, egg, milk, soba (buckwheat noodles) and soy were topping the list as allergens in all age groups [9].

### Intolerances

According to the most frequent intolerances observed in our latitudes, the triggering foods are i) in case of histamine intolerance food with high histamine content or large amounts of biogenic amines (e.g. phenylethylamine in chocolate); ii) in lactose intolerance milk and dairy products, among which fermented or highly ripened products often contain only very small quantities of milk sugar (yoghurt, hard cheese); iii) and in the case of fructose intolerance, fruits, vegetables and juices with a high content of fructose (pears, apples, etc.). Moreover, it should be noted that sorbitol (a sugar alcohol) can intensify the symptoms and should therefore be avoided, while dextrose improves the tolerability to fructose-containing foods. Therefore, food with an approximate equal ratio of glucose to fructose (or more glucose content) and a low content of sorbitol should be chosen (for example pineapple or blackberry, whereas pears and plums have a high sorbitol content).

### Diagnosis

It is also evident that there are differences between females and males during history-taking interviews for diagnosis of allergies and intolerances. Females search for an empathic chairmanship, for an appreciative atmosphere and for good communication with the physician, whereas males directly target the point and describe their complaints. If the treating physician fails to conduct the conversation in a gender-accustomed form, this could lead to inadequate information to establish correct diagnosis as well as bad patient’s compliance.

Moreover, females use another communication style, as shown by a questionnaire-based study about chest pain: linguistic analyses showed that males were more interested in the cause of the chest pain, and that they were observing and describing it in a very concrete manner, whereas females pictured themselves as pain-suffering and described the pain only diffusely [33].

## Management/therapy

### Allergies

The only adopted and most effective strategy to treat food allergy remains strict avoidance of the offending allergen source(s). In this context, one can notice as well a gender-specific difference, as more females adhered to an allergen-free diet (e.g. gluten-free diet 4.8% of all study-participants compared to 2.3% of males in Mexico [7], also 7.4% vs. 4.1% respectively in Colombia) [34]. However, the majority of participants (93.3%) adhering to the gluten-free diet had no physician’s diagnosis of gluten-related disorders [7]. And even though the diet may be justified (because a gluten-sensitivity might be present), it is not really proven by testing and diagnosis that gluten is the trigger of any possible symptoms in these patients adhering to a gluten-free diet.

Gender-specific differences should also be taken into consideration when treating the patients pharmacologically, as hormonal influences, specific metabolism as well as biological-induced fat and water distribution differ in women and men, medication also differs in its effect. The female hormone estrogen is the reason for different metabolism rates in different phases of the menstrual cycle, observed for instance for the anti-allergic, anti-asthmatic medication methylprednisolone [35]. This may require different doses of the same drug at different phases of menstrual cycle.

Focusing on specific immunotherapy, which is not used for food allergy, the long term results of allergic rhinitis treatment appear to be better in women than in men [36]. Referring to side effects, younger female adults showed a higher risk for systemic, mostly cutaneous side effects upon subcutaneous immunotherapy against grass- and ragweed-pollens [37].

### Intolerances

Avoidance of the symptom-triggering food is the cornerstone for treatment of intolerances. However, variable amounts of the offending substance can still be tolerated by the patient, but need to be determined individually. Moreover, enzymes deficient in intolerance diseases are commercially available as food supplements (e.g., lactase, fructose-converting enzyme, and diaminoxidase). Special convenience food products are also available in supermarkets for patients with food intolerances, e.g. lactose-free, low-histamine or gluten-free. A questionnaire has confirmed that women remain the main consumers of these products. These products are also consumed by healthy patients due to assumed health-related reasons [38].

## Causes and influencing factors for gender-specific differences

### Allergies

**Fig. 1:**
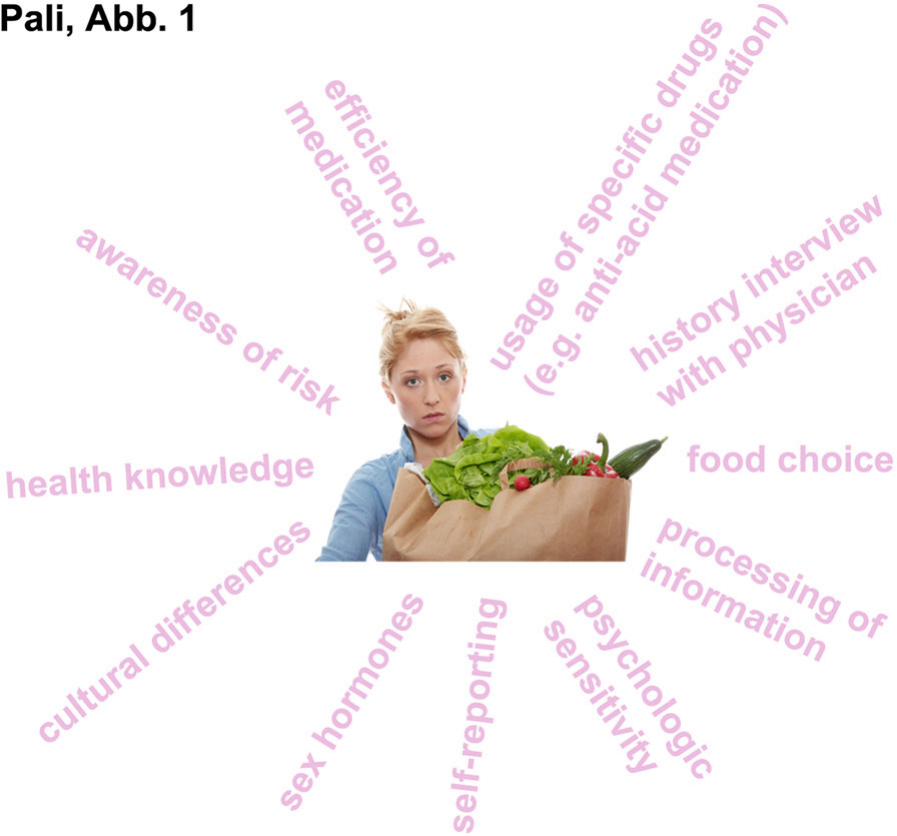
Possible influence factors on higher prevalence of adverse food reactions in girls and women compared to men. (Photo source: Fotolia.com©Piotr Marcinski)

The dissimilar prevalence of adverse food reactions may be multifactorial (reviews [39, 40]), and is related to biological as well as social and cultural gender-related factors (Fig. 1).

The antibody isotype IgG4 is assumed to have a blocking effect against stimulation of mast cells. A possible mechanism is through binding of IgG4 to the allergen, thus blocking the allergen binding to IgE on mast cells. A recent study in 172 healthy adults has now found higher concentrations of IgG4 in males, which may further provide protection against allergy in adult males [41].

The direct effect of sex hormones in allergic diseases has rarely been investigated. It is however well known that women show higher antibody responses against infections and vaccines. Estrogens are a natural enhancer of humoral immune responses (antibody production) and promote autoimmunity; on the contrary, androgens and progesterone (as well as glucocorticoids) physiologically have an immunosuppressive effect [42–45]. Testosterone therefore possibly works against sensitization, whereas estrogen appears to promote clinically relevant allergies [46]. It has consequently been noted that women show variable cycle-dependent T-cell responses [44].

Sex hormone receptors have been found on the surface of lymphocytes, monocytes and mast cells [47, 48]. In general, the pro-inflammatory character of female sex hormones can not only increase the susceptibility for atopy, but also promote delayed Type IV allergic diseases [49]. In addition, endogenous estrogen was found to increase mast cell reactivity [50] and can thereby reduce the required amount of allergen needed to induce allergic symptoms. On the other hand, progesterone works against degranulation of mast cells [51]. Correspondingly, fluctuation in intensity of allergic diseases (especially asthma and eczema) in relation to intake of oral contraceptives, hormone replacement therapy, along the menstrual cycle as well as during pregnancy (high values of DAO improve allergy) was confirmed. Therefore, it’s of great importance to i) inform the patient about these influencing factors and ii) to consider them when prescribing a medication. These hormonal fluctuations possibly also have an influence on the diagnosis of allergy in women and have therefore to be kept in mind during allergy testing. For instance, there is an older paper reporting differences in skin prick test results in allergic as well as healthy women depending on the time point of their menstrual cycle. The reaction to histamine was most intensive on days 12–16 of the cycle, without any differences between the healthy or allergic group, pointing to a clear influence of hormones rather than sensitization on skin prick test results [52]. Therefore, a reasonable and practicable *modus operandi* in our opinion would for instance be to perform diagnostic and follow-up testing within one female patient always at the same time point of her menstrual cycle.

In addition to the genetic predisposition and biological gender-related differences, cultural and environmental factors can affect the sensitization rate and subsequently the prevalence of type I allergies. While girls play mostly in closed rooms, boys more often stay outdoors [53]. Moreover, girls are generally grown up under cleaner circumstances [54]. As a possible result, among children in the age between 5 and 7 years especially girls suffer from atopic eczema.

Different exposure to food allergens — because of varying dietary habits — may influence food sensitization. Men and women (also in healthy population) choose different foods [55–57], for example, while meat and alcohol are usually men’s choices, women are more likely to reach for fruits, vegetables and cereals, probably because women care more for healthy food, while men pay less attention to this issue [57]. The healthier dietary habits of women have also recently been demonstrated in a study, which developed a so-called Eating Choices Index (ECI) score, in which meals *per se* were correlated with their macro- and micro-nutrients contents. In this study, women showed significantly higher ECIs (healthier food) than men [58].

The different food choices can possibly be attributed to different perception of risk, as especially young men tend to have a more optimistic justification; this means, they are convinced that they are not going to experience negative effects when consuming certain types of food (reviewed in [39]). In this context, management strategies and the way of dealing with allergic diseases can also play a role.

The psychological sensitivity of young girls suffering from chronic diseases is generally higher than that of boys suffering from the same diseases (for example epilepsy, asthma or diabetes) [59].

Individual ways of information processing through self-assessment of health condition and a higher rate of reporting food allergies and intolerances (also by parents about their children) between men and women seem influential [60].

Regarding different medications taken by women and men, our own studies about gastric acid-suppressing medications give an example. These drugs also suppress sufficient digestion of proteins, therefore, food may not be digested properly and can consequently lead to a higher risk for food allergy [61, 62]. During pregnancy, the incidence of heartburn, reflux and gastric pain is even higher, making pregnant women more prone to higher consumption of gastric acid-suppressing medications [8, 63].

### Intolerances

It is still unclear, why females suffer more from food intolerances. A possible role of genetic or hormonal effects in transport and enzymatic digestion of dietary carbohydrates or biogenic amines is suspected.

## Conclusion

Food allergy and intolerance can affect both genders; however, they occur more frequently in females after puberty. Females especially suffer more than males from food allergy, food-dependent exercise-induced anaphylaxis and histamine intolerance. In order to confirm the actual prevalence, pathophysiology, influencing factors and consequently the preventive and treatment strategies, it is highly recommended in future studies concerned with different diseases like allergies, to evaluate and analyze the results separately according to gender. Furthermore, multigenerational cohorts will better determine if allergic diseases are more represented in the female sex.

## Abbreviations

AFR: Adverse food reactions
DAO: Diaminoxidase
OAS: Oral allergy syndrome
